# Metabolite signal identification in accurate mass metabolomics data with MZedDB, an interactive *m/z *annotation tool utilising predicted ionisation behaviour 'rules'

**DOI:** 10.1186/1471-2105-10-227

**Published:** 2009-07-21

**Authors:** John Draper, David P Enot, David Parker, Manfred Beckmann, Stuart Snowdon, Wanchang Lin, Hassan Zubair

**Affiliations:** 1 Institute of Biological Environmental and Rural Sciences, Aberystwyth University, Penglais Campus, Aberystwyth, SY23 3DA, UK; 2 BIOCRATES Life Sciences AG, Innrain 66, A-6020 Innsbruck, Austria; 3 Shell Global Solutions (UK), Shell Technology Centre Thornton, P.O. Box 1, Chester, CH1 3SH, UK

## Abstract

**Background:**

Metabolomics experiments using Mass Spectrometry (MS) technology measure the mass to charge ratio (*m/z*) and intensity of ionised molecules in crude extracts of complex biological samples to generate high dimensional metabolite 'fingerprint' or metabolite 'profile' data. High resolution MS instruments perform routinely with a mass accuracy of < 5 ppm (parts per million) thus providing potentially a direct method for signal putative annotation using databases containing metabolite mass information. Most database interfaces support only simple queries with the default assumption that molecules either gain or lose a single proton when ionised. In reality the annotation process is confounded by the fact that many ionisation products will be not only molecular isotopes but also salt/solvent adducts and neutral loss fragments of original metabolites. This report describes an annotation strategy that will allow searching based on all potential ionisation products predicted to form during electrospray ionisation (ESI).

**Results:**

Metabolite 'structures' harvested from publicly accessible databases were converted into a common format to generate a comprehensive archive in MZedDB. 'Rules' were derived from chemical information that allowed MZedDB to generate a list of adducts and neutral loss fragments putatively able to form for each structure and calculate, on the fly, the exact molecular weight of every potential ionisation product to provide targets for annotation searches based on accurate mass. We demonstrate that data matrices representing populations of ionisation products generated from different biological matrices contain a large proportion (sometimes > 50%) of molecular isotopes, salt adducts and neutral loss fragments. Correlation analysis of ESI-MS data features confirmed the predicted relationships of *m/z *signals. An integrated isotope enumerator in MZedDB allowed verification of exact isotopic pattern distributions to corroborate experimental data.

**Conclusion:**

We conclude that although ultra-high accurate mass instruments provide major insight into the chemical diversity of biological extracts, the facile annotation of a large proportion of signals is not possible by simple, automated query of current databases using computed molecular formulae. Parameterising MZedDB to take into account predicted ionisation behaviour and the biological source of any sample improves greatly both the frequency and accuracy of potential annotation 'hits' in ESI-MS data.

## Background

Changes in the overall metabolite composition of living cells (metabolome) reflect a key end point in gene expression and make a major contribution to organism phenotype [1]. Although, no single analytical platform can offer a fully comprehensive survey of the chemical diversity representing the metabolome, continuous improvements in mass spectrometry (MS) instrumentation have allowed development of relatively standardised metabolite profiling or fingerprinting procedures [2]. A fundamental principle of mass spectrometry is the representation of metabolite features in any biological matrix by measurement of the spectrum of signals reflecting the mass to charge ratios (*m/z*) of their ionisation products. One advantage of MS over alternative spectroscopic methods such as Nuclear Magnetic Resonance (NMR) and Fourier Transform Infrared (FT-IR) is the opportunity to putatively annotate directly a spectral component by virtue of its atomic mass. In the context of a metabolomics experiment these 'first pass' annotations may be used to develop hypotheses relating to metabolite identity which are then tested by subsequent, more targeted, analytical chemistry methods. Traditional hyphenated MS *profiling *approaches provide simultaneous detection and quantification of discrete metabolite-derived peaks after chromatographic separation. In gas chromatography MS (GC-MS) a couple of hundred well resolved metabolite peaks are identified where possible by matching their positively charged ion spectrum (following fragmentation by electron impact in the gas phase) and column retention time to those of known standards [3]. GC-MS methods are well established and robust but are limited to analysis only of volatile metabolites. Although derivatisation can increase the volatility of a wide range of metabolite classes such chemical modification further increases the complexity of any annotation process based on atomic mass.

Alternative *profiling *methods utilising liquid chromatography coupled to mass spectrometry (LC-MS) provide sensitive tools for the analysis of a wider range of metabolites with higher polarity, lower volatility and much larger mass range without a need for derivatisation [4]. Resolved metabolite peaks can only be efficiently ionised when outside of the liquid phase under atmospheric pressure. Typical approaches include atmospheric pressure chemical ionisation (APCI) and electrospray ionisation (ESI). In contrast to electron impact, both methods allow 'soft' ionisation with little fragmentation in which a major product may be a pseudo-molecular ion comprising the protonated (+ ve ion data) or de-protonated (-ve ion data) parent molecule.

Peak detection and particularly spectral deconvolution in GC-MS and LC-MS are both technically challenging, time-consuming and very difficult to automate; LC-MS particularly has been hampered by poor analyte peak resolution and retention time variability which confound metabolite peak alignment [5,6]. As a consequence, recent approaches to LC-MS *profiling *have concentrated on the non-targeted quantification of all detected ionisation products above a pre-set noise threshold which are compiled subsequently as several thousand un-annotated variables [7-9]. The variables in this high dimensional data consist simply of an ion mass and its retention time. Such data tables will contain much redundancy as the data acquisition rate of most instruments will be sufficient to generate at least 3–5 scans across each eluting peak. In addition, even with the extra resolving power of ultra high pressure liquid chromatography (UHPLC) a large number of peaks will still overlap or even co-elute in many regions of the chromatogram which demands the use of powerful signal alignment software for data pre-processing.

An alternative approach to LC-MS *profiling *is to generate a metabolite *fingerprint *representation without using the chromatographic dimension in which data variables are simply the detected mass values [10]. In such approaches [2,10-16] the sample may be dissolved in an appropriate solvent and injected directly into the ion source (Direct Injection Mass Spectrometry; DIMS) or infused as a 'plug' flow using a HPLC system without a chromatography column (Flow Injection Electrospray Ionisation Mass Spectrometry; FIE-MS). Data representations may take two forms depending mainly on the accuracy and subsequent resolving power (mass/mass accuracy) of the system mass analyser. In *nominal mass fingerprinting *methods the intensities of all masses from all scans are integrated within pre-defined ranges (mass bins). Linear ion traps with quadrupole (Q) detectors are extremely robust and offer rapid scanning over large *m/z *ranges. However, as full-scan mass resolution is often less than 1000 all signals are binned to the nearest nominal mass value from around *m/z *50 up to as high as *m/z *2000, depending on instrument; under these circumstances metabolites are considered to be mono-isotopic and each mass bin could obviously contain signals derived from several metabolites. Despite these limitations nominal mass fingerprinting methods have attracted considerable interest as a first pass investigation tool as data pre-processing is quick and trivial with minimal likelihood of error and with a short cycle time (typically less than 5 minutes) they are suitable for experimental designs which require a high throughput of samples [10]. Time-of-flight (TOF) and hybrid Q-TOF mass analysers exhibit an average resolving power as high as 10,000 which, with internal calibration, may be sufficient to allow binning of signals in finer mass ranges (0.1 amu). This level of resolution however is not sufficient to distinguish between a large proportion of metabolites of different elemental composition which remain effectively isobaric at the maximum mass resolution achievable. With the advent of ultra-high accuracy mass analyzers such as the Orbitrap (resolution ~100,000) and Fourier Transform Ion Cyclotron Resonance (FT-ICR) analyzer (resolution potentially in excess of 1,000,000) this latter problem is greatly reduced [17-19]. Particularly using FT-ICR-MS instruments there is the opportunity to generate an accurate mass *fingerprint *(often referred to as a *profile *as variables are now considered effectively discrete) of an infused sample extract [20-22]. In such data representations mass 'peaks' are detected in the raw data and the centroid mass intensity calculated. The resolving power and sensitivity of these ultra-high accuracy mass analyzers is such that metabolite signals from molecules containing naturally abundant elemental isotopes (e.g. ^13^C, ^41^K, ^15^N, ^17^O,^34^S and ^37^Cl) are visible in the data [17,18].

The past few years has witnessed a rapid increase in the number of publications describing experiments using particularly electrospray ionisation mass spectrometry (ESI-MS) *profiling *or *fingerprinting *at a range of mass accuracies. This activity has been accompanied by the recognition that the majority of *m/z *variables in most complex biological matrices cannot be matched unambiguously to metabolites of identical elemental composition found in publicly accessible databases [9,19-27]. With the ultra-high mass accuracy achievable using FT-ICR-MS methodology [17,18] this might seem surprising for metabolites with a mass < 500 Da for which the number of isobaric molecules based on calculated elemental composition should be very limited. Several factors contribute to this problem. In the simplest situation, given that many natural metabolites (particularly from plant sources) remain to be structurally identified, the relevant molecules may simply not be present in current databases. A more complex issue stems from the fact that all metabolites have a chance of containing one or more natural isotopes [19,23,24] of constituent atoms (e.g. ^13^C, ^41^K, ^15^N, ^17^O,^34^S and ^37^Cl); metabolite signals thus are rarely present as a single mono-isotopic peak and the spectral isotope distributions often results in measured masses of significant intensity that by chance are undistinguishable (ie not resolvable) from completely unrelated chemistry [18,24]. A final factor relates to the diversity of potential ionisation products generated in the ion source in addition to parental pseudo-molecular ions (i.e. [M+H]^1+ ^and [M - H]^1-^). Thus, in both *fingerprinting *and *profiling *approaches it is common to find adducts with either sample matrix components (e.g. in positive ion data: [M + Na]^1+^, [M + K]^1+ ^and [M + 2Na - H]^1+ ^and negative ion data: [M + Cl]^1- ^and [M + Na - 2H]^1-^) or mobile phase solvents (e.g. [M + NH4]^1+ ^in positive ion data). Additionally, although ESI is traditionally considered a 'soft' ionisation method a number of more sensitive metabolites will rearrange and fragment with neutral loss of common moieties such as water from a hydroxyl group [M - H2O + H]^1+ ^or ammonia from an amine group [M - NH2 + H]^1+ ^or formate from a carboxyl group [M - CO2H + H]^1+^. Clearly, it is possible also that a range of potential ionisation products will contain various combinations of isotopes, adducts and neutral losses. Finally, ionisation products also can carry more than one charge (e.g. [M + 2H]^2+^) or can, through non-covalent interactions, produce stable molecular clusters, for example dimers, in which only one component is charged (e.g. [2M + H]^1+^). The potential to form different ionisation products will be dependent on the structure and physical properties of individual metabolites.

It can be concluded that a key aspect of any variable annotation strategy is to assess the relationships between signals and to base putative annotations on models that best fit all of the data. The presence of isotopic atoms and either loss, or gain, of selected chemical moieties all afford specific changes in accurate mass which mean that the exact mathematical relationships between detected ions are informative. Natural isotope abundances in metabolites reflect the percentages at which isotopes of an element occur naturally in the environment. Thus analysis of isotope relative abundance patterns provide a useful approach to 'filter' unlikely annotations based on molecular formulae calculated from accurate mass measurements [19,23]. Annotation strategies have been reported recently incorporating algorithms interrogating some, but not all, of these atomic mass mathematical relationships [19,22,23,25,26]. In most instances the adopted strategy centred on an analysis of the full spectrum of aligned peaks resolved at ultra-high mass accuracy in the biological matrices under comparison. A less computationally intensive strategy more suitable for high throughput experiments is to focus the annotation exercise on *m/z *signals which make significant contributions to models explaining the metabolome differences between two biologically different sample classes [10,14-16,22,28]. In nominal mass metabolite fingerprint data [10,14-16,28] such experiments make the simple assumption that 'explanatory' metabolites will be represented by several mathematically-related ionisation products (e.g. pseudo-molecular parental ion, ^13^C isotope, salt adducts) which additionally exhibit tight co-variance in a simple correlation analysis; where LC-MS profiling is used ionisation products related to the same metabolite should additionally be found at the same retention time [14,29]. Similar approaches are clearly possible using accurate mass data [7,8,24,27].

It is time consuming and often computationally intensive to pre-process LC-MS profile/fingerprint data to a level where the researcher is absolutely confident of understanding the origin of any ionisation product (i.e. whether signals of interest are parental pseudo-ions, salt adducts, solvent adducts, neutral losses, clusters). This is a particular problem when high-throughput is required or for exploratory analyses of a new biological matrix. An alternative approach described here is the use of a database strategy that will allow searching based on all potential ionisation products that may be predicted to form during electrospray ionisation.

Searching for likely annotation candidates based on accurate mass information in publicly accessible databases is in itself time consuming as individual database coverage of natural chemistry varies and so a comprehensive search requires query of several. Unfortunately, with few exceptions, databases with appropriate metabolite mass information can contain much redundancy, resources for curation are often limited (consequently it is not uncommon to find mistakes relating to mass values, molecular formulae and structure) and some of the entries relate to ionic states, often from interactions with salts. To overcome some of these problems we have developed MZedDB [30] a database which uses an archive in a common format of all metabolite 'structures' derived from several widely used and publicly accessible databases. The value of such an approach to avoid ontology problems was recognised recently by a consortium of yeast researchers [31]. Using a set of 'rules' derived from structural information and physical properties (such as number of H-bond acceptor/donors, number of OH/COOH/NH2 groups, number of acidic H or basic O in molecule) MZedDB will generate a list of potential adducts and neutral loss fragments that are likely to form for each structure and calculates on the fly the accurate mass of every potential ionisation product which provide targets for searches based on accurate mass. Starting with a list of *m/z *signals MZedDB supports a range of manual or semi-automated annotation strategies based on either *m/z *mass or predicted molecular formulae at a range of mass resolutions. In the present article we describe the development of MZedDB and illustrate several typical applications relating to annotation of ESI-MS data which take into account both predicted and measured ionisation behaviour.

## Results and discussion

### Developing MZedDB construction strategy

A major objective of MZedDB was to develop an annotation tool capable of calculating the accurate mass of all likely ionisation products derived from a comprehensive list of natural chemistry represented in a range of web-accessible databases. A first step in this process was to analyse the content of information fields with value for *m/z *annotation and develop a strategy for conversion of the molecular information from targeted repositories into a common format. Once this had been achieved lists could be compiled of all database entries representing molecules with identical structures (i.e. both chemical skeleton and stereochemistry). A preliminary web-search identified many well-populated metabolite databases ranging from chemical class specific (e.g. Lipid Maps [32]), species-specific (e.g. Moto [33]; tomato) and multi-species databases (e.g. Meta-crop [34]) containing information related to measured chemistry. A subset of databases with links to genome sequence data, metabolic pathway representations and in some cases literature provide further information which help annotation decisions related to known or predicted natural chemistry (e.g. KEGG [35], MetaCyc [36], and HMDB [37]). Finally there exist very large repositories, such as PubChem [38] and ChemSpider [39], which contain information on large numbers of metabolites which are not necessarily all of natural origin. Detailed examination revealed that the data fields useful for signal annotation could be divided broadly into four categories: origin, analytical information, physical properties and metabolite relationships (Table 1). All databases had a standard molecular formula for each metabolite entry and the most common structural information sufficient to discriminate between the majority of molecules was clearly a Simplified Molecular Input Line Entry System representation (SMILES), which could also be generated from the more complex IUPAC International Chemical Identifier (InChI) or an MDL Molfile.

**Table 1:** Comparison of data fields useful for LC-MS *m/z *signal annotation in a selection of online databases

**Major Data Class **	**Field Description**	**Databases with field**
**Origin**	Chemical source	H
	Synthesis reference	H, MT, Kp
	Biofluid location	H
	Tissue location	H, Cs
	Biofluid concentrations	H
	Drugs	K, P, Ch
	Synthetic molecules	P, Ch

**Known**	SMILE	H, P, Ch, Cy, MB, Cs
	INCHi	P, Ch, Cs
	Molfile	K, Ch
	H-bond acceptor/donor	P
	Physiological charge	H, P, Ch, Cy, MB, ML, MC, MT, K, Cs, Kp
	Predicted mass	H, P, Ch, Cy, MB, ML, MC, MT, K, Cs, Kp

Analytical information*	Fragmentation	H, MB, Kp
	Measured mass	K, H, P, MB, MT, Kp
	Retention time	ML, MB, MT
	Melting point*	ML, MB, MT, Cs
	LogP*	H, Cs
	H20 Solubility*	H, P, Cs

**Metabolite relationships**	Chemical hierarchy	H, P, Cy
	Metabolite pathways	K, H, Cy, MC
	Reaction Information	K, Cy, MC
	Enzyme Information	K, Cy, MC

**H **= Human Metabolome Database

**MT **= Moto

**K **= KEGG

**P **= PubChem

**Ch **= ChEBI

**Cy **= MetaCyc ,

**MB **= Massbank

**MC **= MetaCrop

**ML **= Metlin

**Cs **= ChemSpider

**Kp **= KNApSAcK

* These data are often predicted from structural information.

**Figure 1:**
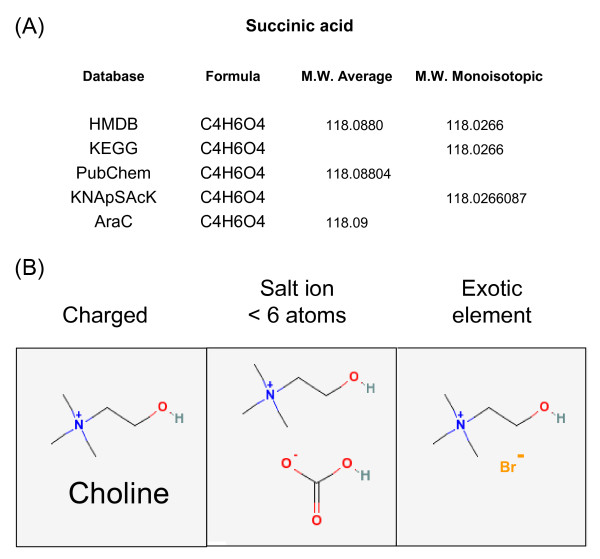
**Metabolite data representations in several web-accessible metabolite databases**. (a) Accurate mass information relating to succinic acid in several large databases (see legend to Table 1 for abbreviations). (b) three structurally diverse entries for choline in PubChem.

Information on atomic mass was much more varied (Table 1 and Figure 1A); for example databases such as MetaCyc (in this case AraCyc) did not provide accurate mass data. Accurate mass information was presented in different databases as either the average molecular weight or mono-isotopic molecular weight, ranging from 4 to 7 decimal places. Annotation success increases more or less linearly with mass accuracy [19]; with Oribitrap and FT-ICR-MS capable of operating at or above 100,000 mass resolution then mono-isotopic mass information down to 4–5 decimal places will be required to optimise annotation success. Additionally, in several databases (particularly the large PubChem and ChemSpider repositories) metabolite information was not always represented as a single neutral charged molecule which will potentially complicate most automated annotation procedures which assume a signal is derived from a single molecular entity composed of pre-selected common atoms (e.g. C, O, N, H, S); an example is shown in Figure 1B of choline which is represented in ionic form on its own, or together with separate common or more exotic salts. Based on this analysis it was decided that a comprehensive coverage of natural metabolites could be achieved by downloading molecular information from the targeted repositories and then processing (see Methods section for details) all chemical entries to remove salts (i.e. keeping the largest component) and to remove molecules with less than 6 atoms or exotic elements. When possible all charged entities were converted to neutral compounds by addition or removal of hydrogen. The processed molecular information was then represented as SMILES, each of which had a unique identifier code in MZedDB and a hyperlink to the entry in the database of origin.

### Development of 'rules' to link metabolite structures represented in MZedDB to potential ionisation products

SMILES, a unique ID and hyperlinks are the only information permanently stored in MZedDB for each metabolite entry accessed from external databases. SMILES can be used to generate a structural representation of the metabolite in question using the Java applet *Jmol *if required. Automated analysis of SMILES following a set of simple 'rules' can be used to detect the presence of specific moieties that are likely to effect potential ionisation behaviour; for example the possession of NH2 or COOH groups which could be lost as ammonia or formate respectively, or the presence, for example, of hydrogen bond acceptor or donor groups which could affect adduct formation. Additionally, the SMILES can be used to create a molecular formula from which an accurate monoisotopic accurate mass can be calculated.

Using information derived from 'rules' concerning likely ionisation behaviour, an accurate monoisotopic mass can be calculated on the fly for all putative *m/z *signals (parental pseudo-ions, neutral losses, adducts, clusters, multiple charged ions) that potentially represent the molecule in question in any metabolite fingerprint or profile. For the whole range of potential ionisation products calculations are based on the formula



in which the *m/z *of the ionisation product (**m/zIP**) of a metabolite with a specific mass (**massM**) relates to the number of molecules involved in the ionisation product (**xM**) divided by its overall charge (**Charge**) plus the mass, including charge and electrons, to be added (or subtracted in the case of neutral losses) to get the final m/zIP (**Add**). The add functions clearly differ depending on the particular ionisation products and a number of mass calculation rules for common adducts and neutral losses are displayed in Table 2. MZedDB is fully flexible in that the ionisation product calculations [40] can be extended for example if a new solvent is used and solvent adducts are expected, or if samples are likely to contain high levels of unusual salts (e.g. bromine or magnesium) or unusual amounts of specific molecules able to form adducts. These lists of calculated accurate masses will provide targets for queries in experiments to annotate *m/z *signals.

**Table 2:** Example default ionisation product mass calculation rules

**Name**	**Charge**	**xM**	**Add**	**RemAt**	**AddEx**	**RemEx**	**Nelec**	**Rule**
[M+]1+	1	1	0				0	Nch = 1
[M+H]1+	1	1	1.007276632		H		-1	Nacc>0 AND Nch = 0
[M+NH4]1+	1	1	18.03382573		NH4		-1	Nacc>0 AND Nch = 0
[M+Na]1+	1	1	22.98922127		Na		-1	Nacc>0 AND Nch = 0
[M+K]1+	1	1	38.96315853		K		-1	Nacc>0 AND Nch = 0
[M-NH2+H]1+	1	1	-15.0119958	NH			-1	Nnhh>0 AND Nch = 0
[M-CO2H+H]1+	1	1	-44.9982027	CO2			-1	Ncooh>0 AND Nch = 0
[M-H2O+H]1+	1	1	-17.0032881	OH			-1	Noh>0 AND Nch = 0
[M-]1-	-1	1	0				0	Nch = -1
[M-H]1-	-1	1	-1.00727663			H	1	Ndon>0 AND Nch = 0
[M+Na-2H]1-	-1	1	20.97466801		Na	H2	1	Ndon>1 AND Nacc>0 AND Nch = 0
[M+Cl]1-	-1	1	34.96940111		Cl		1	Nacc>0 AND Nch = 0
[M+K-2H]1-	-1	1	36.94860527		K	H2	1	Ndon>1 AND Nacc>0 AND Nch = 0

**AddAt: **formula of the atoms to be added to the molecular formula of one M.

**RemAt: **formula of the atoms to be removed to the molecular formula of one M.

**AddEx**: formula of the atoms to be removed to obtain the final IP molecular formula

(e.g. non covalently bound salts and solvent).

**RemEx: **formula of the atoms to be removed to obtain the final IP molecular formula

(e.g. non covalently bound salts and solvent).

**Nelec: **number of electron to be added when calculating isotopic patterns (mass_e _= 0.0005484).

**Rule: **set of rules to be applied on one M.

**Nacc**: number of H-bond acceptor in M.

**Noh**: number of -OH groups in M.

**Ncoo: **number of -COO^- ^groups in M.

**Naci**: number of acidic H in M.

**Nch: **number of charges in M.

**Ndon**: number of H-bond donor in M.

**Ncooh**: number of -COOH groups in M.

**Nnhh: **number of -NH2 groups in M.

**Nbas: **number of basic O^- ^in M.

### pMZedBD architecture and functionalities

**Figure 2:**
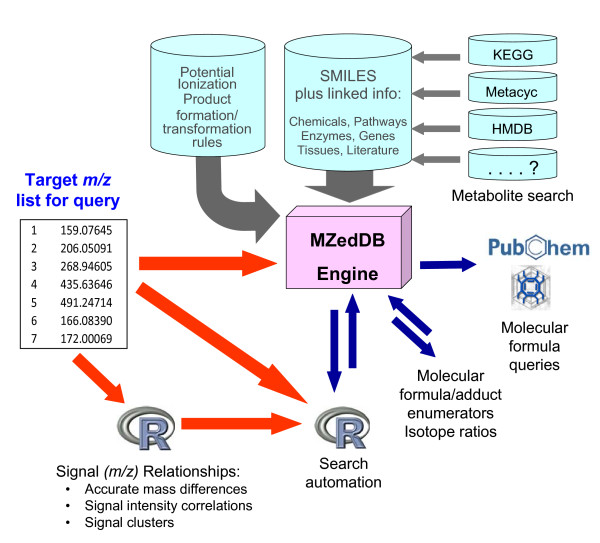
**MZedDB architecture**. Grey arrows represent metabolite information harvesting, processing and hyper-linking for entry into MZedDB; Blue arrows represent MZedDB functionalities; red arrows indicate common query pathways; "....? " indicates that MZedDB can be expanded by integrating data from other databases in the future.

The basic architecture of MZedDB is illustrated in Figure 2. The **Metabolite Search **function can be used to search MZedDB for entries with information in linked repositories related to a specific metabolite name/synonym or a molecular formula or mass (both nominal and/or accurate) of an uncharged metabolite. For example, molecular formulae generated by instrument software can be used for direct query of MZedDB, which provides links to a range of external databases to investigate further details on potential annotations. Alternatively, the MZedDB **MF Generator **offers the opportunity to predict the likely molecular formula of any accurate mass [19,41,42] whilst taking into account the fact that many signals may in fact be adducts, isotopes or neutral loss fragments of a parent molecule. Of more value for *m/z *signal annotation in accurate mass ESI-MS data is the **Putative Ionisation Product **(PIP) function. Searches can be restricted to one, several or all of the external data repositories. Either nominal mass information (with a specific cut off or mass range) or accurate mass information (at any required ppm) can be used to query MZedDB for putative ionisation products (PIPs) derived from known metabolites. Complex queries involving lists of discriminatory masses from data mining experiments (10–50 *m/z*) or processed signals from FT-ICR-MS analysis of an entire biological matrix (many hundreds of signals) can be automated in R using the **R < - > MZedDB function**.

### Parameterisation of MZedDB searches on the basis of potential ionisation behaviour

The parameterisation of MZedDB searches by selection of different PIPs has a significant effect on the outcome of the annotation exercise. For example Table 3 illustrates a typical result when attempts are made to annotate 5 accurate mass signals that were highly ranked, using the Random Forest decision tree algorithm [28], for discrimination between healthy and infected leaves in an interaction between the model grass *Brachypodium distachyon *and the rice blast fungal pathogen [43]. Searching only for [M+H]^1+ ^signals provides potential annotations only for 2 of the 5 ions at < 3 ppm [19], whereas searching for an increasingly diverse range of potential ionisation products provides a small number of hits in MZedDB for all but one of the selected signals (Table 3). The potential identity of metabolites matching the measured exact mass signals are also shown in Table 3, together with their theoretical calculated monoisotopic accurate mass. Using the default setting for potential adducts/neutral losses there are two potential ionisation products ([M+H]^1+ ^and [M+NH4]^1+^) with masses of 159.0764; as ammonium adducts are generally extremely rare in this matrix (unless part of the HPLC mobile phase) it is likely that the [M+H]^1+ ^product is correct. The [M+H]^1+ ^suggestion at 1 ppm for *m/z *172.0007 is phosphoglycolohydroxamate which has a calculated monoisotopic mass of 172.0005, whereas a salt adduct ([M+K]^1+^) of aspartic acid or 2-hydroxysuccinamate are actually exact matches, and indeed much more common metabolites. A sodium adduct of proline betaine is an exact match for *m/z *166.0839 and as it is a naturally charged molecule it will almost certainly predominantly be found as a salt adduct. Several isobaric metabolites are suggested for *m/z *268.9461 when additional adducts were queried after initial annotation attempts using default PIPs found no matches. The importance of considering that major ionisation products could represent adducts or isotopes is highlighted further in Table 4 where the mathematical relationships between all FT-ICR-MS signals in several biological matrices are examined. In spectra derived from biological tissues such as fish liver and human urine greater than 40% of the routinely measured *m/z *signals are likely to be common salt adducts or isotopes. The pre-analysis of a biological matrix can thus help to refine annotation exercises using MZedDB by including only adducts found to be prevalent in the specific matrix. In line with this ethos the MZedDB **Adduct Manipulator **function allows the investigators to expand the generation of possible adducts for a MZedDB entry or a given molecular formula (M->IP), with or without applying adduct formation rules. The reverse transformation (IP->M) can also be performed so that given the molecular formula of a potential ionisation product, all possible formulae of the parent compound can be enumerated.

**Table 3:** Number of putative annotations of FT-ICR-MS signals using MZedDB

Target *m*/*z* (exact mass)	No. of potential hits at 3 ppm				Potential *m*/*z *annotations	Additional *m*/*z *annotations
	[M+H]1+	[M+H]1+ [M+Na]1+ [M+K]1+	Default PIPs	All PIPs	Default PIPS	*All PIPs
159.0764	3	3	8	8	[M + H]1+ *m*/*z *159.0764: 4-methylene-L-glutamine; [M+NH4]1+ *m*/*z *159.0764: 2-aminomuconate semialdehyde OR Gentianaine OR 6-oxo- 1,4,5,6-tetrahydro-nicotinate	-
166.0839	0	3	4	4	[M + Na]1+ *m*/*z *166.0839: Proline betaine	-
172.0007	1	5	5	5	[M + H]1+ *m*/*z *172.0005: Phosphoglycolo-hydroxamate; [M + K]1+ *m*/*z *172.0007: Aspartic acid OR 2-hydroxy-succinamate	-
206.0509	0	0	0	0	No annotation hits	No annotation hits
268.9461	0	0	0	12	No annotation hits	[M + 2K - H]1+ *m*/*z *268.9461: Diketogulonic acid OR 7 other metabolites

Masses highlighted in bold represent perfect accurate mass matches.

* Alternatively, the PIPs selected can reflect prior knowledge of the biological matrix and/or HPLC solvent as in Table 4.

**Table 4:** Prevalence of potential common isotopes and adducts signals in FT-ICR-MS data derived from analysis of extracts of various biological tissues.

**Extract matrix**	**Signal relationship as a percentage of signals as common adducts/isotopes**				
	
	**C13**	**K41**	**M_Na**	**M_K**	**K_Na**
Brachypodium leaf	7.25	2.18	1.97	3.11	3.52
Flounder liver	12.69	2.83	8.98	9.05	9.11
Human plasma	6.98	7.64	4.56	1.68	4.93
Human urine	6.99	4.99	6.85	7.7	28.25
Potato tuber (polar)	4.76	3.87	1.04	3.42	1.64
Potato tuber (non polar)	3.25	0.42	2.54	0.85	1.84

Ideally, MZedDB should be customised intelligently by selection of a subset of PIPs known to be abundant in the matrix under study and searches constrained to databases representing the organism in question. In circumstances where new biological matrices are investigated it may be helpful initially to use the MZedDB default settings in which three parameterisation boxes are automatically 'checked' (Adducts default selection [positive mode]; Apply adducts formation rules; Only include C, H, N, O, P, S) and a search of all database entries is activated from the drop down menu. If negative ion data is under investigation then Adducts default selection should be changed to negative mode. It may be helpful to change the default mass accuracy of 1 ppm to 5 ppm if accurate mass data from Time-of-flight (TOF) or hybrid Q-TOF instruments is used. If large numbers of annotation suggestions are returned then searches can be customised initially by restricting to entries in specific external databases by selecting one or more (using shift key) from the dropdown menu. Further reduction in numbers of annotation suggestions can be achieved by 'unchecking' the default selection boxes and then selecting a customised list of adducts from the pull down menu. Typically, in positive ion data selected adducts would include [M+H]1+, [M+Na]1+, [M+K]1+ and [M-H2O+H]+ and in negative ion data [M-H]1-, [M+Cl]1-, [M+Na-2H]1- and [M+K-2H]1- and [M-H2O-H]1-. Customisation could be extended sequentially as desired to include less common neutral losses (e.g. [M-NH2+H]1+ or [M-CO2H+H]1+), ion clusters (e.g. [2M+K]1+) or ions with multiple charges.

### Guiding m/z annotation decisions by examination of m/z signal relationships

**Figure 3:**
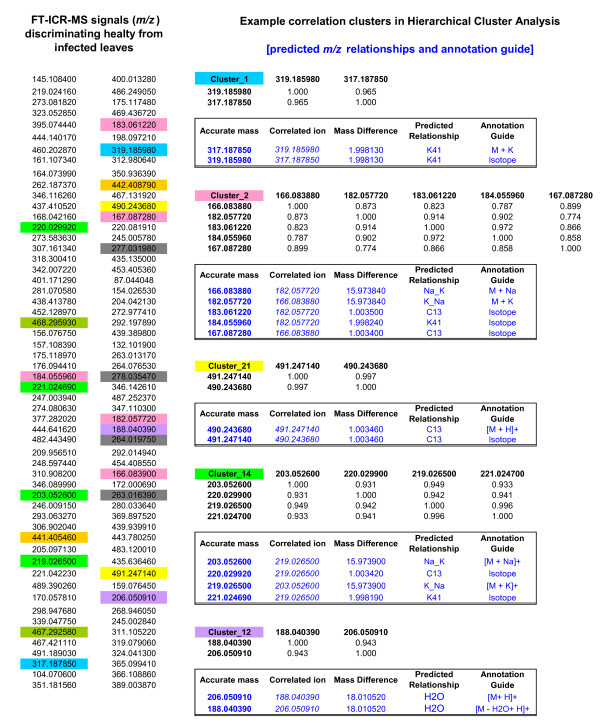
**Correlation analysis and mathematical relationships of explanatory signals discriminating healthy from diseased *Brachypodium *leaves**. The left-hand panel displays the results of feature selection (all < P = 0.001, in descending rank order) in Random Forest classification models comparing FT-ICR-MS spectra of control *Brachypodium distachyon *leaves and plants 96 hours after challenge with a virulent strain of the rice blast fungus. The right hand panel shows example correlation clusters after a hierarchical cluster analysis (HCA) of the metabolome features (shown colour coded) in the left hand panel. The Pearson correlation coefficients are indicated for all combinations of ions in each cluster and the boxes below indicate accurate mass differences, predicted relationships and an annotation guide.

Signals derived from the same parent metabolite will not only exhibit strict mathematical relationships, but, when two relatively similar matrices are compared, the behaviour of related ions should also be correlated in terms of their intensity relationships. The left hand panel of Figure 3 shows a ranked list of the top (*p *= < 0.0001) 'explanatory' positive ion *m/z *signals discriminating healthy from infected *Brachypodium distachyon *leaves 96 hours after infection with the rice blast fungus [43]. A hierarchical cluster analysis revealed that many of the signals fell into small clusters (colour coded) of highly correlated *m/z *(right hand panel of Figure 3). A simple calculation of the accurate mass differences between individual pairs of correlated signals indicates their likely relationships allowing any annotation suggestions to focus on potentially the correct ionisation product. For example, annotation of signals present in Cluster 2 should focus on [M+Na]^1+ ^or [M+K]^1+ ^adducts which are likely to be derived from proline betaine (M = 143.0946). Notably the potential ionisation products with masses of 183.061220 and 167.087280, which are both predicted to be isotopes, had no matches in MZedDB (even at 20 ppm) and so would have been uninformative if pursued further.

**Figure 4:**
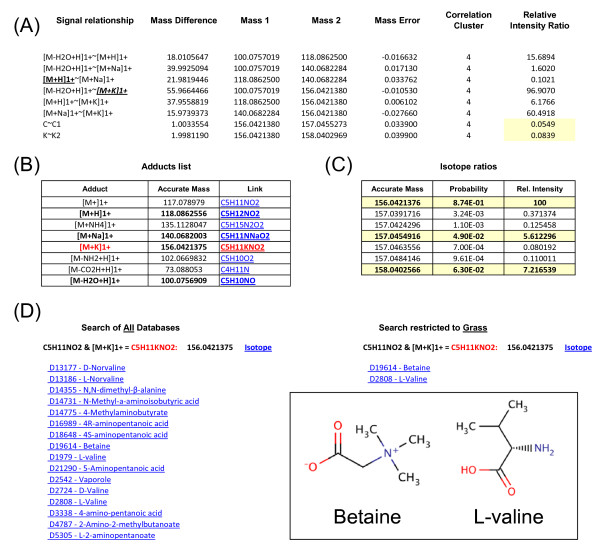
**Investigation of mathematically related signals in a sample matrix**. (A) A typical predicted cluster of mathematically related ions from the full matrix of signals derived from FT-ICR-MS analysis of infected *Brachypodium distachyon *plants with potassium adduct highlighted. Relative intensity ratios of predicted isotopes are highlighted in yellow. (B) Adducts table output following MZedDB PIP search (positive ion) for *m*/*z *156.0421. (C) Isotope ratio predictions table output from MZedDB for *m*/*z *156.0421 with isotopes shown in Figure 4A highlighted in yellow. (D) MZedDB output following a PIP search with the molecular formula C5H11KNO2 of **All **databases (left panel) used to construct MZedDB, or following restriction of search to just **grasses **database entries in KEGG (right panel). Inset shows structure of betaine and valine.

Mass spectrometers capable of high accurate mass measurement often have instrument software dedicated to the identification of molecular isotopes and common adducts in spectra. In most cases only single, simple relationships (such as M_M+1 or M_Na+) are searched for. In addition to a standard Isotope ratio calculator as part of MZedDB development we have developed an 'adduct calculator' (operating in the R environment) which may be tailored to search for masses linked to any number of combinations of adducts, isotopes and neutral losses [40]. Using pre-determined (e.g. 0.001 amu) thresholds arithmetically related signals within a single biological matrix can be tentatively placed into clusters that are potentially all derived from a single parent molecule. Figure 4A demonstrates a typical predicted cluster of mathematically related ions in the full matrix of signals derived from FT-ICR-MS analysis of the model grass *Brachypodium distachyon *and the rice blast fungal pathogen [43]. In this instance the cluster centres on *m/z *156.0421 which is predicted to be a potassium adduct of *m/z *118.0862. MZedDB can be used to query the likelihood that the *m/z *species highlighted in this cluster of signals are predicted to be derived from a single metabolite based on the PIP 'rules' used to construct the database. Figure 4B shows the output of a PIP search (positive ion) for *m/z *156.0421 in which two salt adducts ([M+Na]^1+ ^= 140.0682 and [M+K]^1+ ^= 156.0421) as well as a neutral loss of water ([M-H2O+H]^1+ ^= 100.0756) are predicted to be possible in addition to the parental pseudo-ion ([M+H]^1+ ^= 118.0862). Further investigation using the isotope calculator confirmed that signals *m/z *157.0455 and *m/z *158.0402 (highlighted in yellow in Figure 4C) had the highest probability of being isotopes of *m/z *156.0421 and additionally were present at the correct relative intensities (see last column Table 5A). A PIP search of **All **databases used to construct MZedDB with the molecular formula C5H11KNO2 gave 16 entries corresponding to 10 metabolites; restriction of the database searches to just **Grass **potentially annotated this cluster of signals as being derived from either betaine or valine (Figure 4D).

## Conclusion

In high throughput LC-MS fingerprinting/profiling techniques utilising electrospray ionisation (ESI-MS) it is a common precaution to use analytical instruments at lower than maximal mass resolution to avoid problems with data alignment [21,24]. As a result, *m/z *annotation suggestions may often include a large number of structurally diverse but effectively isobaric candidates when only parental pseudo-ions (i.e. [M + H]^+ ^or [M - H]^- ^*m/z *signals) are considered as ionisation products. Although recent papers have stressed the importance of recognising isotopes to avoid miss-annotation of *m/z *signals in accurate mass LC-MS data [9,18,21,24-26] the present paper shows clearly that ionisation products other than parental pseudo-ions in reality can account for upwards of 40% of the signals in many accurate mass spectra. Against this finding it is unsurprising that many accurate mass *m/z *signals lack annotation 'hits' if structures other than parental pseudo-ions are not included in the searchable databases. Thus we conclude that annotation decisions can be usefully guided by both determining from the outset whether any signal in question is actually an adduct, isotope or neutral loss fragment or indeed any combination of all three. Takahashi *et al*. [27] report the ability to modify a search of the species-specific KNApSAcK database dependent upon the presence of sodium, potassium or ammonium adduct ions derived from the solvent used for sample preparation. Other researchers have downloaded the general metabolite database KEGG LIGAND and calculated the exact mass of 7 possible adduct ions for each entry to provide query targets in a customised database [22]. MZedDB consolidates and expands on this strategy by harvesting SMILES and calculating the accurate mass of a much more comprehensive list of metabolites from a range of databases, as well as including hyperlinks to rapidly access detailed information from entries in the original databases. By restricting MZedDB searches to entries derived from only a selected number of the original databases *m/z *annotations can be refined in a species specific context. An additional advantage of MZedDB centres on the ability to extend annotation searches to include a much larger range of adducts, neutral loss fragments and clusters by implementation (using on the fly calculations) of a simple set of Potential Ionisation Product 'rules' which effectively filter searches to exclude unlikely candidates based on SMILES information and which can be customised in line with any prior knowledge concerning the matrix under analysis (e.g. salt content of tissue, extraction or HPLC solvent). Ultimately, metabolite identity assignment is crucial to derive biological meaning from metabolome modelling experiments and thus data annotation tools such as MZedDB which allow more intelligent searching need to be expanded to cover more comprehensively a wider range of natural chemistry and take into account the characteristics of new biological matrices. For example, to improve the analysis of human urine samples it is anticipated that future implementations of MZedDB will be extended to include any common biotransformations of parent metabolites (e.g. glucuronidation, sulphation, and de-methylation) in attempts to link ionisation products to original metabolites.

## Methods

### Generation of FT-ICR mass spectrometry data

The majority of data used to evaluate the functionality of the MZedDB annotation systems was generated on a Thermo LTQ instrument fitted with a 7-Telsa FT-ICR mass analyser. Extract preparation and basic instrument set up has been described previously [10] but the present study utilised an Advion Nanomate chip-based direct infusion nanospray ionisation to introduce the sample [22]. Typical nanospray conditions comprised 200 nl/min flow rate, 0.5 psi back pressure, and +1.6 kV (positive ion data) or -1.6 kV (negative ion data) electrospray voltage, controlled by Chipsoft software (Advion). FT-ICR-MS parameters included an automatic control gain setting of 1 × 10^5 ^and a mass resolution of 100,000 and data was recorded for 6 min per replicate infusion using Xcalibur software (Thermo Scientific). Blank quality control samples comprising extraction solvents were interspersed at random into the run sequence to monitor instrument performance and detect system peaks. Prior to any statistical analysis the data was log transformed to reduce the chance of the higher intensity peaks dominating in any multivariate analyses.

An FT-ICR-MS data set representing flounder liver extract generated by the SIM-stitching method [44] was kindly supplied by Prof. Mark Viant and Dr Andrew Southam (School of Biosciences, University of Birmingham, UK).

### Data Processing and Statistical Analysis

Data within each biological matrix class (ie leaf tissue, urine, potato tuber etc) were aligned and any peaks not represented in 70% of replicates were removed from the matrix. Two basic types of data sets were available for the project. The first type comprised a population of biological replicates representing a typical 'tissue' class; to provide some diversity the samples included fish liver (flounder), human urine, human blood plasma, plant leaves (*Brachypodium distachyon*) and potato tubers. The second type of samples were characterised by being part of a larger experiment that contained several biologically related sample matrices. For example a series of leaf sections harvested at different time points following infection of the grass *B. distachyon *with the rice blast pathogen [43] provided a complex sample series involving the interaction of two organisms. All sample classes could be used to generate FT-ICR-MS spectra containing thousands of resolved accurate mass signals (full matrix analysis) to provide typical annotation targets. FT-ICR-MS matrices derived from the infected *Brachypodium *samples additionally allowed both a detailed analysis of signal correlation behaviour and provided sample populations for further supervised multivariate data mining [28] in order to identify a small sub-set of 'explanatory' features (*m/z*) able to discriminate between the different diseased states.

All statistical tests were carried out in the R environment using the FIEMSpro metabolomics data analysis package [29] which is web accessible [45]. Explanatory feature selection was performed using Random Forest [15,16,27]. Signal correlation analysis was carried out by the Pearson correlation method and performed on the explanatory *m/z *obtained after feature selection. Hierarchical cluster analysis based on the correlation coefficient was employed to identify the set of clusters, which satisfy some setting, for example, a signal correlation coefficient larger than 0.75. Determining the mathematical relationships between *m/z *is performed in R as described [46]. This code searches for operator predetermined mass differences between measured accurate masses at an adjustable sensitivity (examples of mass difference searches are shown in Figure 4). In theory any mass difference can be searched for providing the operator knows the exact expected mass difference between the measured masses. This process is important to indicate possible isotope signals present in the matrix for which a prediction would not be wanted, and as an indication of the relationship between ionisation products within the matrix.

### Developing MZedDB database

MZedDB is designed as a relational database which is implemented in a MySQL server running on PowerPC 1.8 ghz with 2 GB ram. The web-based interface is publicly available [30]. All calculations were performed within the same environment using Perl and Unix shell commands to generate MZedDB tables and the ChemAxon software suite [47] to manipulate chemical structures. Interaction between MySQL database, user queries, results display and external applications was implemented using PHP. The Isotope Pattern Calculator (IPC v 1.3) [48] was employed to simulate isotopic patterns based on element atomic masses and probabilities. The integrated molecular formula generator [42] is a modified version of the tool available from Tobias Kind [49].

#### Overview of MZedDB table structure

MZedDB comprises a set of several tables containing relevant information from a range of web-accessible databases containing molecular information relating to metabolites (see Table 1 in Results). Due to the lack of a universal ontology for representing metabolite chemical information [31] and the non existence of a standalone platform where metabolite information is stored, our strategy relies on harvesting molecular information from web accessible metabolite databases. This information is then aggregated according to the actual chemical structures contained in each database, rather than using ambiguous annotation based on preferred names, synonyms or even CAS registry number. At the time of writing, only a few metabolite databases have been deposited and regularly updated to PubChem, making the use of its standard chemical information for searching PIP fairly restrictive. Finally, links between entries in different databases were not used because they may either be incomplete or somehow erroneous, irrespective of their level of curation.

#### Generating MZedDB *dbcompound *table

Chemical structures and associated pathway and reactions when available were downloaded from the corresponding websites (see Table 1 in Results section). Molecular structures, database entry ID, names and synonyms were parsed using bespoke Perl scripts. Because of the various formats used to represent molecular structure in each source (e.g. InChI , SMILES  and SDF ) and the inherent difficulties associated with matching chemical information from different chemoinformatics software, we adopted a common SMILES (Simplified Molecular Input Line Entry System) format generated with MolConverter (v. 5.l.4 )[50].

To enable comparisons across databases, we followed a multiple step strategy to clean up each entry. Ambiguities related to salts and complexes were removed by keeping the largest fragment. Structures containing less than 6 atoms, elements other than (C, H, N, O, P, S, K, Cl, Br, F) and ambiguous fragments (e.g. R) were then systematically excluded. A third stage consisted of reducing a major source of discrepancies between databases resulting from the dual representation of neutral and ionic forms of the same entity by removal (positive ion) or addition (negative ion) of a hydrogen atom. Finally, each molecular entity was aggregated according to: (1) Any exact match identification number; (2) A selected name from the list of names given by the original source; (3) Aggregated list of all synonyms; (4) Entry SMILES; (5) Canonical SMILES. Whenever available, stereochemical information was retained during the curation process, giving rise to MZedDB redundancies (e.g. glucose has several entries corresponding to the full or partial description of the configuration of its 5 asymmetric centres). The provision of Canonical SMILES (i.e. representation without specified stereochemistry) allow proposition of alternative entries with similar chemical structure.

#### Other MZedDB tables

Alongside the MZedDB compound table, ***dblinks ***functions to regroup the ID in the primary metabolite database for each MZedDB entry in order to generate dynamic hyperlinks to the original database. The table ***dbmetrule1 ***contains properties of each MZedDB entry that are required to perform the Potential Ionisation Product search. This table encapsulates molecular formula generated from the SMILES and the accurate monoisotopic mass computed to seven decimal places to avoid inaccuracies while rounding at a lower precision level. The remaining columns in ***dbmetrule ***consist of the overall charge and cardinalities of a predefined set of chemical groups necessary to apply adduct formation rule restrictions during Potential Ionisation Product (PIP) search. Metabolic pathway related information is contained in two tables. The table ***reac1 ***regroups the reaction description from each primary database (i.e. identifier, names, enzyme annotations, pathway identifiers and names). The table ***reac2 ***provides links for each MZedDB entry to reactions in which the compound is known to be involved.

### MZedDB search capabilities

As the initial goal of MZedDB was not to provide a chemoinformatics environment for metabolomics, any metabolite-related queries were reduced to elementary searches based on chemical composition, molecular weights and synonyms. A metabolite entry reflects chemical properties relevant to *m/z *annotation and provides links to primary database sources for known pathway/enzymatic information to help biological interpretation. Links to entries with the same skeleton (canonical SMILES) and molecular formula are also provided. A typical output file based on a query for information on pipecolic acid is presented [see Additional file 1]. The user can interactively check its potential ionisation products and verify their exact isotopic pattern distributions to corroborate with experimental data.

Primarily, MZedDB focuses on extensive PIP search capabilities [51] using an extendable list of potential adducts (in both positive and negative ionisation modes) and chemistry based formation rules to avoid impossible or highly improbable PIPs entering the list of potential candidates [40]. For example the possession of an amine group that will allow the neutral loss of ammonia or the presence of strong hydrogen bond donors or acceptors which affect interactions with salts and solvents to generate molecular adducts. As MZedDB allows either a default selection, or user-specified selection (including no rules) of adducts, neutral losses, clusters, charged states and number of charges, only a subset of 'rules' are applied 'on the fly' depending on which options are 'checked' when setting up a query. The end result is that when MZedDB is queried with one or more accurate masses only the masses of parent metabolite derivatives that satisfy the chosen set of PIP rules are available as annotation targets.

The annotation search can be parameterised by defining the level of mass resolution (in ppm), simply an *m/z *range or nominal mass given the truncation value for binning. Further parameterisation of MZedDB is achieved by limiting to metabolites found only in specific organisms, groups of organisms or databases. Databases restrictions are obviously straightforward. Limiting search to metabolites found in specific organisms is achieved by linkage to KEGG information. Additionally, the search can be extended to the more general generation of acceptable PIP molecular formula given the overall charge, the number of non covalently bound units and a mass precision. Potential molecular formula of the parent compound deduced from the PIP table are used to search a local installation of PubChem (to avoid heavy traffic to the server).

## Availability and requirements

• Project name: MZedDB

• Project home page: 

• Operating system(s): Multiple platform (tested on Windows and Mac OS X).

• Programming language: php, MySQL, Perl, C++

• Other requirements: Java 1.3.1 or higher

• License: None

• Any restrictions to use by non-academics: None

## Authors' contributions

JD conceived of the study, and participated in its design and coordination and drafted the manuscript. DE conceived the original MZedDB architecture and table structure. DE, MB and DP were responsible for refining MZedDB query and output functions. DE developed software code for Compound Table construction, the Adduct Calculator, Isotope Calculator and Molecular Formula Generator and implemented the database web interface. MB and DP provided analytical chemistry and mass spectrometry data processing expertise for all stages of MZedDB design and implementation. DP generated and processed FT-ICR-MS data and led the effort to test MZedDB functionality with the participation of SS, HZ and JD. WL and DP carried out statistical analyses and participated in development of software code for investigating mathematical relationships of ESI-MS signals. All authors read and approved the final manuscript.

## Supplementary Material

Additional file 1 **Example MZedDB Metabolite Card**. This document shows an example MZedDB Metabolite Card entry for pipecolic acid. Click here for file
