# Supplementary material for: Metabolite signal identification in accurate mass metabolomics data with MZedDB, an interactive m/z annotation tool utilising predicted ionisation behaviour 'rules'
Source: BMC Bioinformatics. 2009 Jul 21;10:227. doi: 10.1186/1471-2105-10-227 (PMC2721842; doi:10.1186/1471-2105-10-227)
Supplement: Additional file 1 — Example MZedDB Metabolite Card. This document shows an example MZedDB Metabolite Card entry for pipecolic acid. [file 1471-2105-10-227-S1.doc]

**[Additional files](http://www.biomedcentral.com/bmcbioinformatics/ifora/#h1data%23h1data)**

#### Additional file 1

#### File format: DOC

#### Title: Example MZedDB Metabolite Card

Description: This document shows an example MZedDB Metabolite Card entry for pipecolic acid.

#### Metabolite entry:

| Database id | D22652 [Adducts](http://maltese.dbs.aber.ac.uk:8888/hrmet/search/genipres.php?did=D22652&applyrule=1) |
| --- | --- |
| Selected name | Pipecolic acid |
| Synonyms | Pipecolic acid;;L-pipecolate;;()-Piperidine-2-carboxylic acid;;(+/-)-2-Piperidinecarboxylate;;(+/-)-2-Piperidinecarboxylic acid;;(+/-)-Pipecolate;;(+/-)-Pipecolic acid;;(+/-)-Pipecolinate;;(+/-)-Pipecolinic acid;;(.+/-.)-2-Piperidinecarboxylic acid;;(RS)-2-Piperidinecarboxylate;;(RS)-2-Piperidinecarboxylic acid;;.alpha.-Pipecolinic acid;;2-Carboxypiperidine;;2-Pipecolinic acid;;2-Piperidinecarboxylate;;2-Piperidinecarboxylic acid;;2-Piperidinylcarboxylic acid;;a-Pipecolinate;;a-Pipecolinic acid;;acide pipecolique;;acide piperidine-carboxylique-2;;alpha-Pipecolinate;;alpha-Pipecolinic acid;;Dihydrobaikiane;;DL-2-Piperidinecarboxylate;;DL-2-Piperidinecarboxylic acid;;DL-Homoproline;;DL-Pipecolate;;DL-Pipecolic acid;;DL-Pipecolinate;;DL-Pipecolinic acid;;Hexahydro-2-picolinate;;Hexahydro-2-picolinic acid;;Hexahydropicolinate;;Hexahydropicolinic acid;;Homoproline;;pipecolate;;pipecolic acid;;Pipecolic acid free base;;Pipecolinate;;Pipecolinic acid;;piperidine-2-carboxylic acid;;Piperolinate;;Piperolinic acid |
| Accurate mass | 129.078979 |
| Molecular Formula | C6H11NO2 [Search](http://maltese.dbs.aber.ac.uk:8888/hrmet/search/metsearch.php?mform=C6H11NO2) [Isotope](http://maltese.dbs.aber.ac.uk:8888/hrmet/search/isotope.php?molform=C6H11NO2&rellim=1) [Adducts](http://maltese.dbs.aber.ac.uk:8888/hrmet/search/genipres.php?mf=C6H11NO2) |
| Number of atoms | 20 |
| Number of stereocenters | 0/1 |
| Smiles | [OC(=O)C1CCCCN1](http://maltese.dbs.aber.ac.uk:8888/hrmet/search/dispmol.php?smi=OC(=O)C1CCCCN1&nam=Pipecolic acid) |

#### Data source(s):

- HMDB: Pipecolic acid [(HMDB00070)](http://www.hmdb.ca/metabolites/HMDB00070)
- Metacyc: L-pipecolate [(L-PIPECOLATE)](http://biocyc.org/META/NEW-IMAGE?type=COMPOUND&object=L-PIPECOLATE)

#### Known metabolic reactions:

- Metacyc: Small-Molecule-Reactions [(RXN-8166)](http://biocyc.org/META/NEW-IMAGE?type=REACTION&object=RXN-8166)
  - Enzyme(s): [1.5.1.21](http://www.expasy.ch/enzyme/1.5.1.21)
  - lysine degradation V [(PWY-5283)](http://biocyc.org/META/NEW-IMAGE?type=PATHWAY&object=PWY-5283)
- Metacyc: Small-Molecule-Reactions;Chemical-Reactions [(RXN-8161)](http://biocyc.org/META/NEW-IMAGE?type=REACTION&object=RXN-8161)
  - No associated enzyme
  - lysine degradation V [(PWY-5283)](http://biocyc.org/META/NEW-IMAGE?type=PATHWAY&object=PWY-5283)
- Metacyc: L-pipecolate oxidase;Small-Molecule-Reactions [(L-PIPECOLATE-OXIDASE-RXN)](http://biocyc.org/META/NEW-IMAGE?type=REACTION&object=L-PIPECOLATE-OXIDASE-RXN)
  - Enzyme(s): [1.5.3.7](http://www.expasy.ch/enzyme/1.5.3.7)
  - No associated pathway
- Metacyc: L-pipecolate dehydrogenase;Small-Molecule-Reactions [(L-PIPECOLATE-DEHYDROGENASE-RXN)](http://biocyc.org/META/NEW-IMAGE?type=REACTION&object=L-PIPECOLATE-DEHYDROGENASE-RXN)
  - Enzyme(s): [1.5.99.3](http://www.expasy.ch/enzyme/1.5.99.3)
  - No associated pathway
- Metacyc: δ(1)-piperideine-2-carboxylate reductase;Small-Molecule-Reactions [(1.5.1.21-RXN)](http://biocyc.org/META/NEW-IMAGE?type=REACTION&object=1.5.1.21-RXN)
  - Enzyme(s): [1.5.1.21](http://www.expasy.ch/enzyme/1.5.1.21)
  - No associated pathway

#### Related compound(s) with identical skeleton:

- [D23128](http://maltese.dbs.aber.ac.uk:8888/hrmet/search/printmet.php?metid=D23128) - L-Pipecolic acid
- [D23147](http://maltese.dbs.aber.ac.uk:8888/hrmet/search/printmet.php?metid=D23147) - D-Pipecolic acid

#### Related compound(s) with similarity >0.8:

- [D23147](http://maltese.dbs.aber.ac.uk:8888/hrmet/search/printmet.php?metid=D23147) - D-Pipecolic acid - 1.000
- [D23128](http://maltese.dbs.aber.ac.uk:8888/hrmet/search/printmet.php?metid=D23128) - L-Pipecolic acid - 1.000
- [D22648](http://maltese.dbs.aber.ac.uk:8888/hrmet/search/printmet.php?metid=D22648) - d1-piperidine-dicarboxylate - 0.934
- [D19675](http://maltese.dbs.aber.ac.uk:8888/hrmet/search/printmet.php?metid=D19675) - Homostachydrine - 0.882
- [D23678](http://maltese.dbs.aber.ac.uk:8888/hrmet/search/printmet.php?metid=D23678) - 5-hydroxy-pipecolate - 0.864
- [D13187](http://maltese.dbs.aber.ac.uk:8888/hrmet/search/printmet.php?metid=D13187) - (2S)-2-{[1-(R)-Carboxyethyl]amino}pentanoate - 0.827
- [D5306](http://maltese.dbs.aber.ac.uk:8888/hrmet/search/printmet.php?metid=D5306) - (2S)-2-{[1-(R)-carboxyethyl]amino}pentanoate - 0.827
